# Hypoxia in obesity - from bench to bedside

**DOI:** 10.1186/1479-5876-10-S2-A20

**Published:** 2012-10-17

**Authors:** Jianping Ye

**Affiliations:** 1 Antioxidant and Gene Regulation Laboratory, Pennington Biomedical Research Center, Louisiana State University System, Baton Rouge, Louisiana 70808, USA

## 

It is generally accepted that hypoxia is related to sleep apnea in obesity. This concept has been changed since the report of hypoxia response in adipose tissue of obese mice by our group in 2007 [1]. The observation has been confirmed by many laboratories in multiple obesity model systems including mouse and human [2-8]. The adipose tissue hypoxia has been a new concept to explain the adipose tissue dysfunction in obesity [9,10]. It provides a unified answer to all of the pathological changes in the adipose tissue under obesity, such as chronic inflammation, ER stress, leptin expression, adiponectin reduction, adipocyte death, elevated lipolysis and adipocyte insulin resistance [9,10]. Studies suggest that capillary dysfunction occurs during expansion of adipose tissue [11,12], and leads to reduction in adipose blood supply [13], which is responsible for the tissue hypoxia. In this aspect, the adipose tissue dysfunction is a result of local vascular failure in obesity [13]. In addition, the hypoxia-induced inflammation response has beneficial effects in the body. For example, inflammatory response stimulates adipose tissue remodeling [11,14] and promotes energy expenditure to fight against obesity [15,16]. These new insights into the adipose tissue biology suggest that the hypoxia response may be a feedback mechanism in the protection of body against obesity. In translation of this view into clinical setting, it is believed that sleep apnea is also a protection mechanism in the body to maintain energy homeostasis in obesity. It uses the hypoxia response to trigger the onset of multiple protection mechanisms in the body.
